# Community-owned resource persons for malaria vector control: enabling factors and challenges in an operational programme in Dar es Salaam, United Republic of Tanzania

**DOI:** 10.1186/1478-4491-9-21

**Published:** 2011-09-28

**Authors:** Prosper P Chaki, Stefan Dongus, Ulrike Fillinger, Ann Kelly, Gerry F Killeen

**Affiliations:** 1 Ifakara Health Institute, Coordination Office, Kiko Avenue, Mikocheni, PO Box 78373, Dar es Salaam, United Republic of Tanzania; 2 Liverpool School of Tropical Medicine, Vector Group, Pembroke Place, Liverpool L3 5QA, UK; 3 London School of Hygiene and Tropical Medicine, Department of Disease Control, Keppel Street, London, WC1E 7HT, UK

## Abstract

**Background:**

Community participation in vector control and health services in general is of great interest to public health practitioners in developing countries, but remains complex and poorly understood. The Urban Malaria Control Program (UMCP) in Dar es Salaam, United Republic of Tanzania, implements larval control of malaria vector mosquitoes. The UMCP delegates responsibility for routine mosquito control and surveillance to community-owned resource persons (CORPs), recruited from within local communities via the elected local government.

**Methods:**

A mixed method, cross-sectional survey assessed the ability of CORPs to detect mosquito breeding sites and larvae, and investigated demographic characteristics of the CORPs, their reasons for participating in the UMCP, and their work performance. Detection coverage was estimated as the proportion of wet habitats found by the investigator which had been reported by CORP. Detection sensitivity was estimated as the proportion of wet habitats found by the CORPS which the investigator found to contain Anopheles larvae that were also reported to be occupied by the CORP.

**Results:**

The CORPs themselves perceived their role as professional rather than voluntary, with participation being a *de facto *form of employment. Habitat detection coverage was lower among CORPs that were recruited through the program administrative staff, compared to CORPs recruited by local government officials or health committees (Odds Ratio = 0.660, 95% confidence interval = [0.438, 0.995], *P *= 0.047). Staff living within their areas of responsibility had > 70% higher detection sensitivity for both Anopheline (P = 0.016) and Culicine (P = 0.012): positive habitats compared to those living outside those same areas.

**Discussion and conclusions:**

Improved employment conditions as well as involving the local health committees in recruiting individual program staff, communication and community engagement skills are required to optimize achieving effective community participation, particularly to improve access to fenced compounds. A simpler, more direct, less extensive community-based surveillance system in the hands of a few, less burdened, better paid and maintained program personnel may improve performance and data quality.

## Background

Cities and large towns are regarded as some of the most favourable environments for sustainable public health development programs because of their relatively well educated, readily accessible populations, with access to information, governance and social infrastructure [1,2]. Nevertheless, many vertically-organized public health programs have had limited success because they fail to engage the community members in their planning and implementation [3,4]. It has consistently been elucidated that these obstacles are not due to a lack of medical, epidemiological or ecological technical competences, but rather a lack of knowledge on how to achieve the effective coverage through the widespread involvement of the communities in question [5,6]. This has led many public health programs to adopt community participation as a fundamental basis for effectively and efficiently delivering interventions by overcoming resource limitations and maximizing intervention acceptability [7-9]. It is widely acknowledged that community involvement can improve intervention coverage, efficiency and effectiveness, as well as promote equity and self-reliance [4,10,11]. However, although there is general consensus about the benefits of community involvement on public health development, the strategies adopted are widely variable depending on the socio-political context, institutional culture and the nature of community organization [12,13]. It is thus possible, for the same strategy, to produce quite different effects; where there is a high level of social solidarity, communities will actively involve themselves, whereas where there is not the response things may be more passive [5,6]. While community mobilization is perceived as a potentially powerful, unexploited resource, and a means to appropriately and efficiently meet basic health needs [7,13,14], comprehending and converting the rhetoric of community participation into reality remains a great challenge in public health [6,15,16]. This is especially true in the fragmented urban societies that are characterized by heterogeneous needs and mobile human populations.

The participation of communities in vector-borne disease control is context-dependent. [4,9,17,18]. The degree of community involvement is determined by the type of disease targeted, available intervention options and the endemicity level [6,9,16,19]. The constituent activities of vector control can be implemented either intermittently, as with insecticide residual spraying (IRS) or insecticide-treated bed nets (ITN) distribution campaigns, or routinely, as is the case for larvicide application or transmission surveillance [14,20-22]. In either case, community engagement is essential as both interventions must be integrated into everyday activities and domestic or local environments. Furthermore, because vector control requires a comprehensive coverage, in addition to active daily participation, communities require administrative support. Thus strategies which combine extensive mobilization of community-based labour [20,22,23] with vertical management structures--embedded within pre-existing local government structures and public health systems--may enable affordable, scalable and sustained community compliance while maintaining rigorous standards [17].

A number of review papers have identified these key determinants of successful community participation in public health programs [7,16,24]. In the case of vector-control, meaningful, substantive collaboration between communities and institutional support experts has successfully lead to the sustainable abatement of malaria and other vector-borne diseases [2,14,19,25]. Malaria control through larviciding or through larval habitat reduction are intervention options with which considerable successes have been recorded both historically and very recently [26-34]. It is notable that the most prominent recent large-scale [22] example relied upon extensive community involvement through vertical management systems to overcome the complex spatially variable mosquito larval ecology of relevant vector species and the resulting need for rigorous, labour-intensive foot searches for larval habitats [20,22,29]. Such expert-community interactions often rely upon relatively few skilled personnel--carefully chosen from within local communities--who shoulder the responsibility for implementating and communicating to the community at large, so as to maximize compliance and effective coverage [20]. It is widely accepted that well-chosen health personnel selected from within a community are more likely to gain community confidence [5,19], and are therefore more efficient as behaviour change agents to achieve the desired impact [18]. It is therefore essential for programme managers to consult the relevant communities prior to implementation, in order to understand and anticipate local political forces, cultural and social interactions, as well as expectations [4,13], as these will influence participation among not only recruited individuals, but also the entire community. To understand the degree to which people will participate, it is important first to understand whether or not people will comply with the interventions. Moreover, if people do participate, it is important to understand how they interpret and value their involvement in the program over time [35].

The Urban Malaria Control Program (UMCP) in Dar es Salaam, Tanzania has been initiated by the Dar es Salaam City Council as a pilot program to develop sustainable and affordable systems for larval control, as part of routine municipal services [14,22,23,27,36-41]. The goal of the UMCP is to evaluate the effectiveness of a large-scale, community-based larval control program to reduce malaria transmission. The UMCP implements weekly application of microbial larvicides (*Bacillus thuringiensis *var. *israelensis (Bti) *and *B. sphaericus (Bs) *to all potential breeding habitats, and delegates responsibility for these routine mosquito control and surveillance to community members, referred to as Community-Owned Resource Persons (CORPs) [22].

Studies have revealed that even members of the most marginalized communities could be well protected from mosquito bites if given access to relevant knowledge, skills and resources [3,42-44]. The UMCP aims to address this capacity deficit by building partnerships between communities and malaria control experts. All UMCP activities are fully integrated into the decentralized administrative system in Dar es Salaam, in accordance with the local government structures introduced under the Local Government Act number 8 of 1982 as a response to adopting the Alma Atta Declaration (1978) [45], thus operating on all five administrative levels of the city.

The Health and Environmental Sanitation Committees at the ward and street levels are responsible for community participation in the health system, mobilizing resources from within communities, notably casual labour, and ensuring that hygienic conditions are maintained--which includes monitoring the performance of individuals in health-related projects [45]. These committees typically consist of an average of eleven members. Despite their longstanding existence, little is known about how these committees function in practice or the extent of their impact on public health service delivery. One of the challenges faced by these committees is a lack of clarity in their terms of reference, particularly in relation to the extent and nature of their interaction with the community base.

This paper characterises the strengths and weaknesses of a recent effort to reinstate larval source management in Dar es Salaam, implemented by community members through UMCP. The central aim of this study is to generate a better understanding of the role that the CORPs play within this programme, and the operational pre-requisites for these to contribute effectively in terms of representing the community voice, mobilizing the required resources and achieving the desired impact. By investigating the CORPs--their demographic characteristics, their reasons for participating in the UMCP, and their work performance--this study outlines how communities can become responsible for malaria control and, more broadly, how the audience of public health is realized within UMCP.

## Materials and methods

### Study area

Dar es Salaam is Tanzania's biggest and economically most important city with a population which already exceeded 2.5 million inhabitants in 2002, estimated to reach 3.3 million in 2010, living within an administrative region of 1400 km^2 ^[46,47]. The city is divided into three municipalities, namely Kinondoni, Temeke and Ilala, and these municipalities are further divided into a total of 72 wards. The study site comprised the 15 wards (5 per municipality) with 614 000 residents [46] included in the Dar es Salaam UMCP, covering an area of 56 km^2 ^[14,22,27,48]. All UMCP activities are coordinated by the City Medical Office of Health, and are fully integrated into the decentralized administrative system of Dar es Salaam. UMCP operates on all six administrative levels of the city: the city council, the three municipal councils it oversees, the fifteen wards chosen from those municipalities--containing 67 neighbourhoods referred to as *mitaa *in Kiswahili (singular *mtaa*, meaning literally street)--and more than 3000 housing clusters known as ten-cell-units (TCUs), each of which is subdivided into a set of plots corresponding largely to housing compounds. The main tasks of the 3 upper levels are programme management and supervision, whereas actual mosquito larval surveillance and control is organized at ward level and implemented at the level of TCUs and their constituent plots. In principle, a TCU is a cluster of 10 houses with an elected representative known as an *mjumbe*, but typically comprises between 20-100 houses in practice [40]. Between 2004 and 2009, the UMCP implemented regular surveillance of mosquito breeding habitats as a means to monitoring effective coverage of aquatic habitats with microbial larvicides [22]. Surveillance was done through a community-based [23] but vertically-managed delivery system [22]. The cross-sectional surveys described here to evaluate routine surveillance activities were conducted between June 2007 and January 2008.

This study used a mixed-method research design, combining qualitative and quantitative approaches [49,50].

### Routine programmatic larval surveillance by community-based personnel

Community-Owned Resource Persons (CORPs) were recruited through local administrative leaders, particularly including Street Health Committees. They were remunerated at a rate of 3000 Tanzanian shillings (2008: US$ 2.45) per day, through a casual labour system formulated by the municipal councils of Dar es Salaam, for a variety of small-scale maintenance tasks such as road cleaning and garbage collection [14,23]. Over 90 larval surveillance CORPs were actively employed by the UMCP during the time of the survey, with each CORP assigned to a defined area of responsibility comprising a specific subset of TCUs within one neighbourhood. These subsets of TCUs were initially allocated based on local knowledge of habitat abundance, difficulty of terrain and geographic scale, and subsequently refined through detailed participatory mapping of the study area, so that each CORP was responsible for an average area of approximately 0.6 km^2 ^[40]. All CORPs worked under the oversight of a single ward-level supervisor and followed a predefined schedule of TCUs, which they were expected to survey on each day of the week. In wards where larviciding was taking place, the schedule of TCUs visited by the surveillance CORPs followed one day after they were visited for the application of microbial larvicides, by a separate set of larval control CORPs [22]. By doing so, indicators of operational shortcoming, such as the presence of late-stage (3^rd ^or 4^th ^instar) mosquito larvae, could be reported and reacted to fast enough to prevent emergence of adult mosquitoes. This system was designed to enable routine mosquito habitat surveillance and larviciding, with the specific objective of allowing timely interpretation and reaction to entomologic monitoring data.

### Qualitative preliminary assessment of community-based larval surveillance

Using structured participatory observation, one of the investigators (PPC) initially conducted three weeks of unscheduled guided walks with 23 of the surveillance CORPs. These CORPs were nominated by their respective ward supervisors after the investigator reported to their office unannounced in the morning. The investigator did not pre-inform the CORPs nor did he reveal his role and independent status at any time before or during the visit. Both the investigator and the chosen CORPs would leave the ward office and survey TCUs that the CORPs were expected to survey according to their normal predefined schedule for that particular day [22], returning later to report to the ward supervisor. At this stage, the survey was led by the CORPs and the investigator followed passively, observing and recording how CORPs conducted their routine larval habitat surveillance and prepared their daily reports for submission to the ward supervisor. Specifically, the following six key questions guided observation on whether individuals adhered to the set standard operating procedures [22]:

(1) Did CORPs follow their schedule correctly?

(2) Were all TCUs and plots visited?

(3) Were fenced compounds entered, and if not, why not?

(4) How were habitats recorded?

(5) How were habitats searched for larvae?

(6) How did CORPs interact with residents?

In cases of observed shortcomings in the operational practices of the CORPs, or any additional opportunities for improved implementation of their duties, the investigator provided the CORPs with informal advice. This approach was intended to maintain an open, non-authoritative relationship of the investigator with the CORPs, allowing the investigator to observe and understand the operational challenges faced by the CORPs and the program as a whole. Informal appraisal of these observations was used to design a quantitative survey described as follows [39].

### Quantitative cross-sectional evaluation of community-based larval surveillance

A total of 173 TCUs from neighbourhoods distributed across all 15 wards were randomly selected from the list of TCUs in the UMCP study area. A total of 64 CORPs were responsible for these selected TCUs. The investigator accompanied the relevant CORPs during the survey through each TCU one day after their scheduled routine surveillance of that TCU and implemented his own larval habitat surveys following the standard operating procedures [22]. At this stage, the visits remained unannounced but the investigator's role was revealed. The investigator conducted a comprehensive search of each plot for potential breeding habitats and then searched each of those for mosquito larvae following standard operating procedures [22]. First, the larval survey data sheet filled by the CORP on the previous day was examined. Then the presence of every reported wet habitat was verified, and each one was re-examined for the presence of larvae or pupae. Then any additional habitats that had not been detected by the surveillance CORPs were identified and examined for the presence of larvae. All data for the follow-up survey of the investigator were recorded using standardized forms adapted from those provided to the larval surveillance CORPs [22,39]. The proportion of wet habitats reported by CORPs was compared to the total number of all potential habitats by the investigator to establish the detection coverage, whereas detection sensitivity was established by comparing the proportion of habitats which contained larvae that were reported by the CORP with that reported from the investigator's survey.

Additional information was collected regarding the presence or absence of a fence around a plot and whether or not a particular TCU was targeted with larvicide application at the time that it was surveyed. Lastly, records were taken regarding evidence of lack of familiarity of a CORP with the specific TCU and plot. Unfamiliarity was assumed if the CORP was not readily able to find his or her way around the TCU or plot, when plot boundaries could not be clearly defined, or when residents of the plot were unable to recognise him/her as a regular visitor to the area [39]. At the end of each visit, a structured questionnaire was administered to collect data regarding the individual characteristics of the CORPs, including gender, age, place of residence and recruitment history (Additional file 1).

### Data analysis

The results from the participant observation during the guided field walks with the CORPs were subjected into content analysis to identify the main themes. Our interpretation of themes articulated in interviews is supported by a comparative ethnographic research on community participation in larval control projects in the Gambia [51]. The fully pre-coded numeric forms with interview responses were entered and analyzed using SPSS 16.0. Generalized estimating equations were fitted to determine the influence of the various factors upon the proportion of wet habitats (detection coverage) reported by CORPs and the proportion of habitats which contained larvae that were reported to be occupied by the CORP (detection sensitivity). The factors included were clear knowledge of project goal, frequency of field visits by supervisor, where the individual CORPs lived, relationship with the residents, by whom individuals were recruited, and time spent to get to the field. While all observed habitats were included in the model fits to assess detection coverage, only those found by the CORPs and reported to contain larvae by the investigator were considered in the denominator of the models to assess detection sensitivity. The detection of the wet habitat or its larval occupancy by the CORP were each treated as the binary outcome variable which was fitted to a binomial distribution with a logit link function. CORP identity was treated as the subject variable and an exchangeable correlation matrix chosen for the repeated measurements which were distinguished by habitat identity as the within-subject variable.

## Results

### CORPs' demographic characteristics

Overall, 64 CORPs, of whom 36 were male and 28 female, were surveyed. All of the respondents initially received work-related training at recruitment, organized by the program staff. This primarily involved field/practical training to develop basic skills for the identification of different types of breeding habitats, aquatic-stage mosquitoes and operational skills such as community engagement and obtaining access to private plots. In addition to field training, 83% (53) of the interviewed individuals had also attended seminars, 61% (39) had received relevant reading materials and 58% (37) received both. Those respondents who had only attended primary education numbered twenty six (41%), with the remaining majority having secondary education. Approximately half (52%, 33) of these CORPs were between 20 and 29 years of age, while 28% (18) were between 30 and 39 and the remaining 20% (13) were 40 and above. Individuals' age correlated positively with the length of time they had spent working for UMCP (r^2 ^= 0.327, *P *= 0.008). A third (31%, 20) of the respondents had been with the program for one year or less. Four-fifths (81%; 52) of the respondents stated they had no other source of income. All of those with another source of income (19%, 12) were involved mainly in petty trading. Of the interviewed CORPs, 34% (22) reported to have formally or socially recognized positions within their respective Community Health and Environmental Committees at either the ward or neighbourhood level. Of those interviewed, 9% (6) had previously worked in similar vector control programmes in the past [52]. The majority (59%; 38) of the interviewed CORPs reported spending between six and seven hours in the field each day, while 22% (14) spent between eight and nine hours a day, and 19% (12) spent four to five hours in the field.

The initial quantitative evaluation results showed a substantial improvement in the detection and correct identification of breeding habitats [39], compared with previous prototype systems [23]. The majority of the CORPs exhibited basic competence in identifying and reporting malaria vector breeding sites: almost three thousand aquatic habitats were recorded during the survey, of which 66.2% (1963) were detected by the 64 CORPs [39], implying that the majority of them had at least a basic understanding of how to identify mosquito breeding sites. As previously described, the observed detection sensitivity for mosquito larvae was consistently low [39].

### Contextual determinants of detection coverage identified through the guided walks

Initial observations and analysis of the interview data from the guided walks with the individual CORPs and supervisors suggested that, despite their enthusiasm for the work, the community-based staff wished to be consulted more in decisions made concerning the working conditions of the program. The major concern expressed was the unfair distribution in work between the CORPs and other UMCP staff at program management levels (Table 1). CORPs cited a number of incidents that had happened to some of their colleagues or themselves, which they considered illustrative of the lack of understanding of the working conditions by the higher operational levels within UMCP administrative hierarchy. During the discussion, one respondent emphasized in particular the failure of administrators to take into account the daily needs of CORPs and the consequences this had for their wellbeing (Table 1).

**Table 1:** Assorted responses from the interviewees illustrating the main contextual factors influencing their routine performance

	CORPs	Ward & Municipal Supervisors
**Community relations**	*I encounter problems entering some of these houses. For example, here lives a white man, he keeps snakes and dogs. I have not been able to go in because the security guards had advised me not to, even though I can see from here that there is a swimming pool and tires but I could not do anything. Maybe the project leaders should assist us in educating these people because I have shared this with my supervisor but she could not help me*. *Sometimes you get to a fenced house so you knock at the gate. First comes the house girl and she asks what you want. You explain that you need to go inside to look for breeding places and she might tell you just wait. So you stand there waiting for minutes. Then a boy comes and he asks you to explain again. If you are lucky they will let you in, otherwise you will be told the house owners are not here so come later or tomorrow. This takes a lot of time, so sometimes we do not bother to go there*.	*For me as a supervisor, I find it easy to work here because I belong to this ward and I am a member of the environmental committee, so I have no problem working with people. (Ward Supervisor)* *Some of the CORPs they have had previous experiences, with UNICEF or other projects, so they know how to approach people and inform them. Others are inexperienced and the moment they run into problems, they stop the work and give up (Municipal Coordinator)*

**Views on UMCP work**	*We are responsible for the project - we are working all day out in the field. The supervisors are not out here in the field and they receive a far greater amount...if we were valued as part of the project, like the supervisors, it would make the job easier for us*. *The work I do is hard, but it is a good project... I have come to know the community members. We are all hoping there will be more opportunities and we will continue to do this work*.	*The CORPs who work with us are very good, the problem is not many stay with us for long - it is very difficult work, they go and the training is lost. We need to be careful in our selection, ones that have experience and will have an easier time, it is no good when they come and go (Municipal Coordinator)*. *This project has worked best where the community is most involved. If we give power to the Mtaa leaders to select, coordinate and fund larval control it will be sustainable. (Municipal Coordinator)*

**Motivation to participate**	*I feel like this is the only way out for me, because at least I get assured of being paid at the end of the month...* *I need at least some time off. I have to rest for at least a week and, at the same time, use that opportunity to meet my relatives. But the way things are, if I go on leave for just a day I will not be paid, and I do not want that to happen because I need that money*.	*This has been a good project and has made a large impact on the community. We are all thinking it should be continued, though we cannot be assured what will happen in the next years. We are now all working well together, we can only hope that the project is taken up permanently (Ward Supervisor)*.

**Working conditions**	*I remember there was one CORP, who was working here, but he got sick and so for days he could not go to work. He was very sick but the project did nothing to help him until his relatives came to take him to their home. He unfortunately had to go for treatment. So even if you get sick, you still have to find a way to at least get to work so that you can get the money for that day, because we need money and the project has no budget for treatment*.	*I think being a supervisor is a tough job, because you not only have to look at your own work, but also make sure that even those under you are doing the right job There is so much to be done because I have to split my time between going to the field to see what they are doing and check the reports that I receive because I do not trust some of them. Now that we are applying the larvicide, it is even tougher because I have to check on the two teams and yet if you look at what we are being paid it is very little unlike our fellow inspectors [municipal level]. They do little but they get paid twice what we get. (Ward Supervisor)*.

Most CORPs explained that though they are regarded as volunteers working on a part time basis, the work is so demanding and exhausting that it takes up most of their day and they become too tired to do anything else that could contribute to their livelihood (Table 1). There was a high degree of job dissatisfaction tied to the amount of remuneration they received per working day, which was not perceived as being proportional to the working hours and effort invested. A recurring challenge to the comprehensive habitat surveillance and achieving sufficient coverage was gaining access to fenced compounds [39]: One CORP complained that supervisors, while sympathetic, were also not capable of crossing these socio-economic barriers. Most interviewees continually emphasized how these drawn-out social negotiations exacerbated the workload.

Across the interviews, the most salient enabling factor was the CORPs' ability to relate positively with the residents in those areas. Being able to relate to home-owners was generally associated with having worked previously with the Community Health Committees. A third of the CORPs (34%, 22) and their supervisors repeatedly mentioned that having a recognized formal role within these respective bodies made their work easier by enabling effective communication with the residents. This was especially true in relation to accessing enclosed, often-guarded compounds and removing container-type habitats, such as tires, within those plots.

Much of this points to limited access, motivation among staff, and compliance among residents with project activities, and partly explains how and why individual CORPs were recruited into UMCP. The fact that almost half of all aquatic habitats were located within fenced plots [39] makes access an even more serious obstacle to intervention coverage. One-fifth of all aquatic habitats were recorded in plots with which CORPs clearly appeared unfamiliar, and over 90% of these were located behind fences [39].

It cannot be fully ascertained that the role of the investigator was successfully withheld from the CORPs in all cases and their supervisors probably represent the most likely source of such knowledge. This may have influenced their working practices while with the investigator, so the practices reported here may well be positively biased to some degree.

### Determinants of aquatic habitat detection coverage

The aquatic habitat detection coverage levels varied significantly between wards (*P *< 0.001), probably reflecting individual geographic variation and ward-level variation in the quality of supervision [39]. The probability of detecting and recording breeding habitats by CORPs was significantly reduced if the CORP could not clearly explain the overall goal and activities of the programme (Table 2). Individuals' clarity of understanding of the programs' objectives positively correlated with the time length they have worked for the program (Pearson correlation, r^2 ^= 0.472, *P *< 0.001). This implies that, as individuals spend longer times with the programme, they become more competent, knowledgeable and accurate advocates for the project within their communities and areas of responsibility. However, staff turnover was a major problem within UMC, as almost one-third (31.2%, 20/64) of the CORPs interviewed reported to have been working with the program for less than a year. The implied high turnover rate is obviously problematic for a labour-intensive program relying on experienced personnel to realize effective implementation and community engagement.

**Table 2:** Factors associated with mosquito larval habitat detection coverage

**Interviewee response**	***Proportion of respondent CORPs*** ***%(a/64)***	***Proportion of habitats detected by CORPs*** ***%(n/N)***	**OR [95%CI]**	**P**
				
***Clear knowledge of project goal and advocacy level ***	*NA*	*NA*	NA	0.002
Complete	*59 (38)*	*70.0 (1281/1829)*	1.00^b^	NA
Incomplete	*41 (26)*	*59.4 (675/1136)*	0.596 [0.403,0.880]	0.009
				
***Who individuals were recruited by ***	*NA*	*NA*	NA	0.004
Community local leaders	*79 (50)*	*68.4 (1625/2375)*	1.00^b^	NA
Project administrative staff	*22 (14)*	*56.1 (331/590)*	0.660 [0.438,0.995]	0.047
				
***Perceived relationship with the residents***	*NA*	*NA*	NA	0.028
Very supportive	*64 (41)*	*62.7 (1068/1703)*	1.00^b^	NA
Reasonably supportive	*36 (23)*	*70.4 (888/1262)*	1.627 [1.053,2.515]	0.028
				
***Time spent to get to the field ***	*NA*	*NA*	NA	0.011
Less than or equal to quarter an hour	*73 (47)*	*65.0 (1477/2273)*	1.00^b^	NA
Above quarter but less than half an hour	*17 (11)*	*78.5 (350/446)*	1.943 [0.965,3.912]	0.063
More than half an hour to one hour	*9 (6)*	*52.4 (129/246)*	0.522 [0.288,0.946]	0.032

The probability that a wet habitat was detected by the CORPs was modelled with a binary distribution and logit link function using Generalised Estimating Equations (GEE) treating clarity and advocacy level, recruiting level, relationship with the residents and the time individuals used to get to the field as potential predictors (exluded factors included: where individuals lived (P = 0.997) and frequency of field visits by supervisor (P = 0.892))

^a ^number of CORPs

CI: confidence interval

OR: Odds ratio

^b ^the reference group for the particular variable

N: the number of wet habitats found during the cross-sectional surveys

N: the number of wet habitats found by CORPs during their routine habitat survey,

NA: Not applicable

Larger areas of responsibility probably increased the amount of time that individual CORPs spent to get to work and search for breeding habitats (Table 2). Consequently, there was a 47.8% reduction in habitat detection coverage among individual CORPs who reported spending an average of half an hour or more to get to their scheduled TCUs (Table 2). However, it is less clear why CORPs that reported to be spending between 15 minutes and half an hour appear to achieve an almost two-fold higher detection coverage. We attribute this observation to either a spurious model fit or to other unknown determinants or covariates of detection coverage, and cannot comment further.

Habitat detection coverage differed significantly among CORPs, depending on who had recruited them into the program: Detection coverage was a one-third lower among individuals that had been recruited directly through programme staff rather than through local community leaders (Table 2).

Furthermore, the reported degree of support provided by residents to interviewed CORPs demonstrated strong influence on the observed habitat detection coverage. Though less uniformly defined, coverage was 63% higher in areas where the CORP reported residents were reasonably--rather than very--supportive of the program. Based on our own observations in the field, we interpret this pattern to imply that CORPs' reports of community supportiveness reflect a measure of honesty among program staff, with answers of "very supportive" probably being exaggerated in most cases (Table 2).

### Determinants of larval-stage mosquito detection sensitivity

As previously described [39], overall detection sensitivity of larvae was very low among the surveyed CORPs (Table 3). As was the case for habitat detection coverage, and presumably for the same reasons, larval detection sensitivity was considerably better among CORPs, reporting that residents were reasonably rather than very supportive (Table 3). Furthermore, detection sensitivity for both Anophelines and Culicines was dramatically lower among CORPs that were not living within their areas of responsibility (Table 3), regardless of whether they lived within the same or external wards. The reductions in Culicine detection sensitivity were statistically significant, and those for Anophelines approached significance (Table 3). However, when the two groups of CORPs living outside areas of responsibility were pooled together, a statistically significant reduced detection sensitivity for Anophelines (OR [95%CI] = 0.25 [0.084, 0.774], *P *= 0.016] and Culicines (OR [95%CI] = 0.26 [0.092, 0.740], *P *= 0.012) was recorded among CORPs in this group, compared to those living within areas of responsibility. More frequent field supervision than the standard recommendation of once per week was associated with reduced culicine detection sensitivity among respective CORPs, presumably because these were known by the supervisor to be poor performers (Table 3). Correspondingly, less frequent field visits than the recommended once per week by the supervisor appear to be associated with more competent CORPs with a threefold increase in culicine detection sensitivity (Table 3). Although no statistically significant influence on anopheline detection was apparent, presumably because this was generally very low, so the number of observations was also low. There was over twofold increase in detection sensitivity among the less frequently visited CORPs (Table 3).

**Table 3:** Factors associated with Anopheline and Culicine detection sensitivity by individual CORPs

**Interviewee response**	**Proportion of respondent CORPs^a^**	**Proportion of Anopheline-positive habitats found by CORPs^b^**			**Proportion of culicine-positive habitats found by CORPs^c^**		
			
	**%**	**%(n/N)**	**OR[95%CI]**	**P**	**%(n/N)**	**OR[95%CI]**	**P**
							
***Relationship with the residents***	**NA**	**NA**	**NA**	**< 0.001**	**NA**	**NA**	**0.041**
Very supportive	64 (41)	10.0 (2/20)	1.00^c^	NA	74.1 (209/282)	1.00^c^	NA
Reasonably supportive	36 (23)	36.4 (28/77)	4.26[2.111,8.597]	< 0.001	60.0 (51/85)	2.77[1.043,7.342]	0.041
							
***Frequency of field visits by supervisor***	**NA**	**NA**	**NA**	**0.400**	**NA**	**NA**	**0.016**
More than once a week	16 (10)	17.4 (4/23)	1.02[0.208,5.036]	0.977	44.2 (19/43)	0.55[0.217,1.377]	0.200
Once a week	61 (39)	36.4 (20/55)	1.00^c^	NA	70.7 (159/225)	1.00^c^	NA
Less than once a week	23 (15)	31.6 (6/19)	2.54[0.580,11.082]	0.216	82.8 (82/99)	3.24[1.016,10.312]	0.047
							
***Where the individual CORPs lived***	**NA**	**NA**	**NA**	**0.098**	**NA**	**NA**	**0.013**
Within area of responsibility	44 (28)	35.7 (15/42)	1.00^c^	NA	77.3 (126/163)	1.00^c^	NA
Within ward of responsibility	31 (20)	31.0 (9/29)	0.30[0.079,1.129]	0.075	71.5 (88/123)	0.24[0.078,0.765]	0.016
Outside ward	25 (16)	23.1 (6/26)	0.24[0.037,1.471]	0.122	58.0 (47/81)	0.21[0.057,0.740]	0.015

^a ^proportion of respondents out of the overall 64 CORPs interviewed

^b ^out of those habitats that were recorded as wet by the CORPs during their routine surveys

The probability of mosquito larvae detected by the CORPs modeled with a binary distribution and logit link function using Generalized Estimating Equations (GEE) excluding time spent to get to the field (P = 0.608), Who individuals were recruited by (P = 0.521) and clear knowledge of project goal (P = 0.654).

^c ^Reference group for particular variable, CI: confidence interval, CORPs; Community-owned resource persons

N: the number of habitats that were reported wet by CORPs during routine habitat surveys and contained larvae during the cross-sectional surveys

n: the number of habitats where CORPs found larvae during their routine habitat surveys,

NA: Not applicable

## Discussion

This study used both qualitative and quantitative methods to explore the perspectives of CORPs and their respective supervisors about the management of UMCP, particularly employment conditions and community engagement practices. The results suggest that there are important differences in perceptions of participation and its associated intervention effectiveness, between the program management levels and CORPs.

Although the UMCP actively involved and depended on CORPs in the routine implementation of breeding habitat surveillance, there appeared to be significant limitations in the employment system with regard to how these human resources were identified, mobilized and maintained. The fact that individuals' ability to detect breeding habitats was reduced when program staff instead of local leaders recruited CORPs emphasizes the need to enforce the policy of local government ownership and control of the recruitment process. It has been demonstrated clearly that most appropriate and effective personnel for implementing community-based interventions are resident community representatives, carefully chosen through the local government leadership. The results confirm the findings of others [5,19] regarding the importance of engaging the resident communities in health development programs.

Overall these results outline a picture of mediocre performance and imply an urgent need for equipping these community personnel with skills to effectively communicate and engage the whole community [53]. Within the UMCP surveillance system at that particular time, more priority was placed on technical larval surveillance and larvicide application skills, with inadequate emphasis on the capacity to interact and communicate. It is therefore important that while training needs to focus on improving technical skills, especially the ability to detect and classify larvae [39], increased emphasis should also be placed on improving individuals' communication skills to enable them to interact more extensively and effectively with the rest of the community. In other words, sensitization has to go beyond mere transfer of knowledge and must seek to optimize community support and engagement for sustainable program effectiveness. This confirms the findings from another study [53] conducted within the UMCP, which focused on resilience-building processes and emphasized the vital role of improved communication among stakeholder communities and the program staff for effective malaria vector control.

A prerequisite for mosquito control programs focusing on larviciding in urban areas is having access to all locations where mosquito breeding takes place. This includes fenced plots and other areas with restricted access for the public, and thus requires substantive and open collaboration between stakeholders. Such collaboration could be achieved by enhancing access to knowledge and information among the various stakeholders at all levels. The fact that habitat detection coverage was higher among CORPs recruited by the local government leadership and the detection sensitivity was generally lower among CORPs residing in areas away from their areas of responsibility suggests one very clear recommendation: Community based personnel should be recruited through the existing community structures such as the community health committees and work only where they live. Furthermore, the recruitment process of the community personnel needs to critically consider the heterogeneity and mobility of the human population in the specified environment, and the socioeconomic and political influences that are likely to shape the level and extent of community participation. Moreover, existing and influential local committees need to be fully integrated, as these are likely to dictate levels of community involvement. It cannot be reasonably expected of city or municipal level staff to fully understand or manage such complex and subtle issues at the fine scales at which implementation occurs, so these tasks must be consistently devolved to the local level.

Moreover, perhaps less extensive but better controlled community-based surveillance with fewer supervisors who are better paid, motivated and retained could improve the quality of data obtained through such community-based surveillance systems. This view can be supported by the supervisor's opinions as expressed in the quotes above of the results section. Following this survey, the UMCP has since been restructured accordingly, with habitat surveillance reduced to a sample of about 6% of TCUs each week. Furthermore, this responsibility is now exclusively allocated to better paid ward supervisors who are no longer overburdened with excessive data collation from numerous CORPs. They are now unambiguously responsible for implementing surveillance in the field themselves in an average of five TCUs per week which are randomly chosen and another five which they choose at their own discretion. It remains to be proven that such changes will yield improvements in these performance indicators and, ultimately, increased epidemiological impact. The results of this study provide a baseline and outline useful indicators with which such systems interventions can be assessed and understood.

## Conclusion

Resident larval surveillance field staff--recruited from within the intervention areas and by the respective local governments instead of the programme management--appear to be most suitable for achieving high breeding site detection coverage and larvae detection sensitivity. Moreover, local governments, and resident CORPs appear ideal for mobilizing the essential resources and the necessary community support for establishing sustainable malaria vector control systems. Improved employment conditions, communication and community engagement strategies--as well as engaging the local health committees in recruiting individual program staff--are crucial factors for achieving effective community participation, and consequently epidemiological impact.

## Competing interests

We declare that none of the investigators has any conflict of interest. None of the funders had any role in the evaluation design, data collection, analysis, interpretation, drafting of the manuscript or decision to publish.

## Authors' contributions

PPC led the design and implemention of the study, data analysis and wrote the manuscript. AK and SD supported the design and implementation of the study. UF, KK and GFK designed and implemented the larviciding system. GFK supervised all aspects of the study design, implementation, data analysis and drafting of the manuscript. All authors have read and approved the final manuscript.

## Supplementary Material

Additional file 1 **Structured questionnaire**. At the end of each visit, a structured questionnaire was administered to collect data regarding the individual characteristics of the CORPs, including gender, age, place of residence and recruitment history. Click here for file
