# Supplementary material for: Community-owned resource persons for malaria vector control: enabling factors and challenges in an operational programme in Dar es Salaam, United Republic of Tanzania
Source: Hum Resour Health. 2011 Sep 28;9:21. doi: 10.1186/1478-4491-9-21 (PMC3204271; doi:10.1186/1478-4491-9-21)
Supplement: Additional file 1 — Structured questionnaire. At the end of each visit, a structured questionnaire was administered to collect data regarding the individual characteristics of the CORPs, including gender, age, place of residence and recruitment history. [file 1478-4491-9-21-S1.PDF]

Municipality: [ ], Ward: [ ], Mtaa: [ ], TCU: [ ], [ ], [ ], [ ], [ ]

Interviewer: ____ **Date: [/ /]**

Identification

Use the following codification: I=Ilala, T=Temeke, K=Kinondoni

Use the following codification:

ILALA	KINONDONI	TEMEKE
1-Buguruni	1-Magomeni	1-Azimio
2-Ilala	2-Mikocheni	2-Keko
3-Kipawa	3-Mwananyamala	3-Kurasini
4-Mchikichini	4-Mzimuni	4-Miburani
5-Vingunguti	5-Ndugumbi	5-Mtoni

Yes=1 No = 2, No answer = -99

CORPs' questionnaire

1. Gender of the respondent F= 1, M= 2 [ ]

2. How old are you in years? [ ]

3. What is your education level?
   Did not attend school at all = 1
   Standard seven = 2
   Form four = 3
   Form six = 4
   No answer = -99 [ ]

4. Did you hear of Urban Malaria Control Program, before you started working for it? [ ]
   Yes=1, No= 2, No answer= -99

5. Do you know what it stands for?
   Not at all = 1
   Partially = 2
   Yes completely = 3 [ ]
   No answer = -99

6. How did you first get to know about UMCP?
   Meeting = 1

Workshop = 2
Media e.g. newspaper, radio, television = 3
From a friend or neighbor = 4
Other = 5, please specify………………
No answer = -99 [ ]

7. Could you please describe the objectives of the program?
(Interviewer tick as relevant from the list)
[ ] control of cholera
[ ] help the citizens to clean their environments
[ ] control of Malaria
[ ] control of filariasis
[ ] control of mosquitoes
[ ] other specify……………..

8. How long have you been with the programme **[ ]**
1-5 months =1
6-12 months =2
13-18 months =3
More than 18 months =4
No answer = -99

9. How did you join the programme? **[ ]**
Chosen by street leaders = 1
Chosen by project administrative staff = 2
Chosen by ward supervisor = 3
Other = 4 Specify……………………………………
No answer = -99

10. When you joined the program did you receive training? [ ]
Yes=1, No= 2, No answer = -99
(*If* ***Yes,*** *go to Q 11, if* **No** *go to Q.16)*

11. From whom did you receive your initial training?
(Mark all that apply)
[ ] Project staff from city level
[ ] Municipal coordinators
[ ] Project Inspector
[ ] Ward supervisor
[ ] A fellow CORP
[ ] No answer

12. From whom did you receive your subsequent training?
(*Mark all that apply*)
[ ] Project staff from city level

[ ] Municipal coordinators
[ ] Project Inspector
[ ] Ward supervisor
[ ] A fellow CORP
[ ] No answer

13. What type of training did you get?
Mark all that apply
[ ] seminar/workshop
[ ] field/site training
[ ] reading materials (e.g. brochures, field guide books, leaflets ect.)
[ ] other, specify
[ ] No answer

14. How often do you receive training now days?
I never receive any further training =1
Less than once a month =2 [ ]
Once a month =3
Once a week =4
More than once a month =5
No answer =-99

15. With the different types of training you are receiving how would you rate them in terms of
usefulness for your job performance,
(1=very poor, 2=poor, 3= moderate, 4= good, 5= very good)
Seminar/workshop [ ]
Reading materials [ ]
Field/on site trainings [ ]
Other [ ]

16. Would you like to have more training? [ ]
Yes=1, No= 2, No answer = -99

17. How many hours do you spend on UMCP activities per day? [ ]

18. How often do you get accompanied by your supervisor in your field work?
Not at all = 1
Less than once per month = 2
Once per month = 3
Once per week = 4 [ ]
More than once per week = 5
No answer = -99

19. How often do you get visited by your inspector?
Not at all = 1

Less than once a month = 2 [ ]
Once a month = 3
Once per week = 4
More than once per week = 5
No answer =-99

20. Do you have any other income generating activities besides UMCP? [ ]
Yes=1, No= 2, No answer= -99
(If Yes, go to qn.21, if No go to qn.23)

21. What kind of activities
(*Mark all that apply*
[ ] Farmer
[ ] Laborer
[ ] Informal sector
[ ] Business
[ ] Fisher
[ ] Government or formal sector employment
[ ] Other, please specify:………………………………………..
[ ] No answer

22. On average how many hours per day do you spend on those activities? [ ]

23. Is your home
Outside the ward you are working =1
Within the Ward but outside the *Mtaa* you are working =2 [ ]
Within the *Mtaa* but not your area of responsibility = 3
Within your area of responsibility where you work as a CORP for the UMCP = 4

24. For how long have you been staying in that house/place?
Less than six months =1
6-12 months =2
More than 1year but less than 5 years =3 [ ]
Five years or more =4
No answer = -99

25. How long do you take to travel from your home to reach the ward of your activity?
1-15 minutes =1
16-30 minutes =2
31-60minutes =3 [ ]
More than one hour =4
No answer = -99

26. How long do you take to travel from ward offices to reach the specific area of your activity?

1-15 minutes =1
16-30 minutes =2
31-60minutes =3 [ ]
More than one hour =4
No answer = -99

27. How would you describe the relationship with community members in the area of operation
towards your activities for the *UMCP*.

They are actively disruptive/hostile =1
They don't accept nor support =2
They are accepting but not supportive =3 [ ]
They are reasonably supportive =4
They are very supportive =5
No answer =-99

28. How would you describe the relationship with *TCU* and Street leaders in the area of operation
towards your activities for the UMCP.

They are actively disruptive/hostile =1
They don't accept nor support =2
They are accepting but not supportive =3 [ ]
They are reasonably supportive =4
They are very supportive =5
No answer =-99

29. How do you rate your relationship with the UMCP ward supervisor?

Very poor =1 good=4
Poor= 2 very good=5
Reasonable=3 No answer= -99

30. What are reasons for?

a)Reasons for negative perceptions

Mark all that apply

He/She is paid more [ ]
Not supportive [ ]
Rarely goes to the field with the CORPs [ ]
Any other [ ] please specify…………
No answer [ ]

b) Reasons for positive perception

Mark all that apply

Very supportive [ ]
He/she is coming from same ward [ ]

His/her living standard matches yours [ ]
He/she frequently accompanies you to the field [ ]
Any other [ ] please specify…………
No answer [ ]

31. Have you worked with any similar programs, if so please describe your impression of the standard of services each provided.

Yes=1, No= 2, No answer= -99 [ ]

Very poor =1 poor = 2 Average=3
Good=4 Excellent=5

Care International	[ ]
Water Aid	[ ]
JICA	[ ]
Plan international	[ ]
World Vision	[ ]
IMPACT	[ ]
……………..	[ ]
……………..	[ ]
